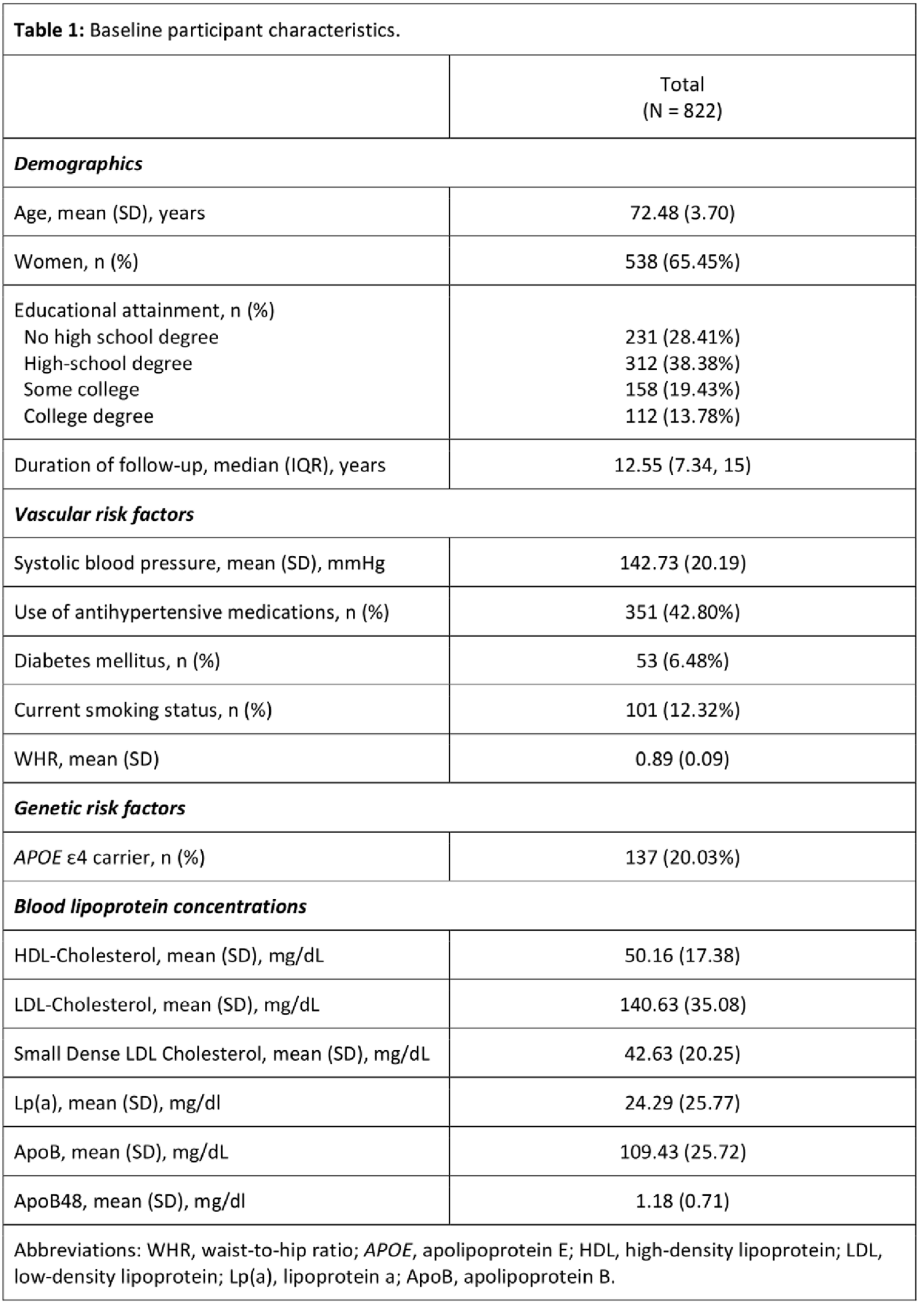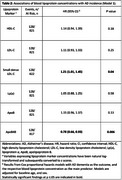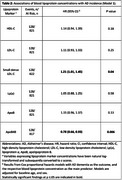# Blood Lipoprotein Concentrations and Risk of Alzheimer's Disease. The Framingham Heart Study

**DOI:** 10.1002/alz70856_102148

**Published:** 2025-12-25

**Authors:** Sokratis Charisis, Sophia Lu, Jesus D Melgarejo, Claudia L Satizabal, Ramachandran S Vasan, Alexa S Beiser, Sudha Seshadri

**Affiliations:** ^1^ Glenn Biggs Institute for Alzheimer's & Neurodegenerative Diseases, University of Texas Health Sciences Center at San Antonio, San Antonio, TX, USA; ^2^ Boston University, Boston, MA, USA; ^3^ Institute of Neuroscience at the University of Texas Rio Grande Valley, Harlingen, TX, USA; ^4^ South Texas Alzheimer's Disease Research Center, Harlingen, TX, USA; ^5^ The Framingham Heart Study, Framingham, MA, USA; ^6^ University of Texas Health San Antonio, San Antonio, TX, USA; ^7^ Department of Population Health Sciences, University of Texas Health Sciences Center, San Antonio, TX, USA; ^8^ Boston University School of Public Health, Boston, MA, USA; ^9^ Boston University School of Medicine, Boston, MA, USA

## Abstract

**Background:**

Proteins involved in lipoprotein metabolism could lie in the causal pathway between cardiovascular health and Alzheimer's disease (AD). The strongest body of evidence exists for Apolipoproteins (Apo) E and J; however, other lipoproteins may be involved. To further explore the physiological links between cardiovascular health and AD risk, we studied the relationships between various blood lipoprotein concentrations and AD incidence in a community‐based sample.

**Method:**

This analysis included 822 participants ≥60 years of age without prevalent dementia from the Framingham Heart Study. Concentrations of high‐density lipoprotein cholesterol (HDL‐C), low‐density lipoprotein cholesterol (LDL‐C), small dense LDL‐C (sdLDL‐C), lipoprotein a (Lp(a)), apolipoprotein B (ApoB), and the ApoB isoform ApoB48 – a specific marker of chylomicron metabolism – were measured by immunoturbidimetric assays and ELISA kits in blood samples obtained from 1985 to 1988. Participants were under surveillance for incident AD until 2020. AD diagnosis was based on standard clinical criteria. The relationships between blood lipoprotein concentrations and AD incidence were explored using Cox proportional hazards regression adjusting for baseline age and sex (Model 1). Additional models were further adjusted for education, vascular risk factors, and *APOE* genotype (Model 2).

**Result:**

Mean baseline age (standard deviation) was 72.5 (3.7) years; 538 (65.5%) participants were women (Table 1). Over a median (interquartile range) of 12.55 (7.34, 15) years of follow‐up, 128 participants (99 women) developed AD. A standard deviation unit (SDU) increase in ln(sdLDL‐C) concentration was associated with a 21% increase in the risk for incident AD (hazard ratio‐HR [95% CI] = 1.21 [1.01, 1.45]), while a SDU increase in ln(ApoB48) concentration was associated with a 22% decrease in the risk for incident AD (HR [95% CI] = 0.78 [0.66, 0.93]) – Model 1 (Table 2). In Model 2, the association with sdLDL‐C remained in the same direction but did not reach statistical significance, likely due to the smaller sample size (Table 3).

**Conclusion:**

Lower sdLDL‐C and higher ApoB48 concentrations were associated with a reduced risk for AD. These findings highlight a potential role of blood lipoprotein markers in AD risk stratification and of lipid modification strategies in dementia prevention.